# Skyrmion Dynamics in a Double-Disk Geometry under an Electric Current: Part Two

**DOI:** 10.3390/nano12213793

**Published:** 2022-10-27

**Authors:** Sebastián Castillo-Sepúlveda, Javier A. Vélez, Rosa M. Corona, Vagson L. Carvalho-Santos, David Laroze, Dora Altbir

**Affiliations:** 1 Grupo de Investigación en Física Aplicada, Facultad de Ingeniería, Universidad Autónoma de Chile, Avda. Pedro de Valdivia 425, Providencia 7500912, Chile; 2 Departamento de Polímeros y Materiales Avanzados: Física, Química y Tecnología, Universidad del País Vasco, UPV/EHU, Paseo M. Lardizabal, 3, 20018 San Sebastián, Spain; 3 Donostia International Physics Center, 20018 San Sebastián, Spain; 4 Departamento de Física, CEDENNA, Universidad de Santiago de Chile, Avda. Víctor Jara 3493, Santiago 9170022, Chile; 5 Departamento de Física, Universidade Federal de Viçosa, Avenida Peter Henry Rolfs s/n, Viçosa 36570-000, MG, Brazil; 6 Instituto de Alta Investigación, Universidad de Tarapacá, Casilla 7D, Arica 1000000, Chile

**Keywords:** magnetic skyrmions, spin torque nano-oscillators, skyrmion–skyrmion interaction, dynamics

## Abstract

Using numerical simulations, we studied the dynamics of two skyrmions nucleated in a double-disk structure. Depending on the geometry and the electric current, different regimes for the dynamical behavior of the skyrmions were obtained. Our results evidence that there are four main dynamic regimes depending on the geometry and current: stagnation points, oscillatory motion, and two types of skyrmion annihilation: partial and total. Our findings are explained as a result of the different forces that skyrmions are subject to and are shown in a state diagram of the dynamical states that allow an adequate understanding of the associate phenomena.

## 1. Introduction

The concepts of topology have been widely used to study several emerging phenomena and particle-like structures in condensed matter physics [1,2,3,4,5]. When considering magnetic systems, topology arguments are used to characterize skyrmions, consisting of swirling spin textures whose stability is ensured by topological protection. This topological protection can be theoretically described by an integer number known as the skyrmion number [6], formally defined by

(1) $$Q=\frac{1}{4\pi }\int d^{2}\vec{x}\;\vec{m}\cdot \left(\frac{\partial \vec{m}}{\partial x}\times \frac{\partial \vec{m}}{\partial y}\right),$$

where $\vec{m}$ is the normalized magnetization. Thus, unless extra energy is injected into the system, a skyrmion belonging to a topological class cannot be continuously transformed into the ground state of a skyrmion belonging to another topological class or another quasiparticle having a different skyrmion number. In particular, in the case of planar skyrmions with Q=1, one can highlight three structures, the bimeron [7,8,9,10], and Bloch and Néel skyrmions [11,12]. Although these three magnetization patterns have the same skyrmion number, bimerons do not exhibit circular symmetry [8,13], while Bloch and Néel skyrmions do. Bloch skyrmions present a swirling structure, similar to a magnetic vortex, and two regions with antiparallel magnetization pointing out-of-plane, the central core and the external border. The Néel skyrmion exhibits the same two regions with out-of-plane magnetization, but instead of having a swirling texture, it presents a radial in-plane magnetization. Therefore, from a theoretical point of view, Bloch skyrmions have their magnetic moments rotating around tangential planes (normal to the radial direction). In contrast, in the Néel-like skyrmions, the magnetic moments rotate in the radial planes [14]. It is worth noticing that although the stabilization of skyrmions in thin magnetic systems is ensured by Dzyaloshinskii–Moriya interactions (DMI) [15,16], it was recently shown that frustrated exchange interactions [17,18,19], dipolar fields [20,21], and curvature effects [22,23,24] may also be responsible for skyrmion nucleation in ferromagnets.

Due to their strong stability and their low current-driving motions [25], magnetic skyrmions have strong potential for applications in logic computing, racetrack memory devices [26], and in general magnetic information [27,28]. The behavior of skyrmions in the presence of external stimuli is also considered for possible applications. For instance, the motion of a skyrmion under the action of magnetic fields or alternating electric currents could be used as resonators [29,30]. In addition, when a time-dependent spin-polarized electron current is injected into a magnetic layer hosting a skyrmion, a torque is created, allowing the skyrmion to rotate around its axis, opening the possibility of producing a spin torque nano-oscillator (STNO) [31,32]. However, using skyrmion for STNO demands a profound knowledge of the skyrmion rotation properties, which depends on the geometry of the system and the frequency of external stimuli [31,32,33,34]. Therefore, several studies have analyzed skyrmion rotation under different geometric and magnetic constraints. For example, Jin et al. [35] reported that the potential created by the inclusion of an annular groove on the surface of a free layer of the STNO yields geometric confinement of the skyrmion, making it rotate with a precession frequency more than six times higher than when no annular groove is included. Furthermore, if the skyrmion moves in a circular STNO under a spin-polarized current, its motion is toward the disk center in a counter-clockwise spiral trajectory [36]. In this case, the skyrmion accelerates at the borders, diminishing its velocity when it is close to the nanodisk’s center.

Since the skyrmion motion in an STNO device strongly depends on the system geometry, the absence of circular symmetry in nanodots yields new phenomena. For example, the analysis of one skyrmion dynamic lying in an asymmetric disk as a function of the geometry and the current showed two different regimes, depending on geometry parameters. Above a threshold value of the geometric parameter defining the nanodisk asymmetry, the skyrmion exhibits a precessional motion with a geometry-dependent radius and frequency [37]. Below such a threshold value, the skyrmion precession converges towards non-centrosymmetric stagnation points [37], confirming the strong influence of geometry on the skyrmion dynamics. Such an impact becomes even more evident if we consider a system composed of two interconnected nanodisks. Indeed, in a work previously published by our group [38], we showed that depending on the current density and the disk interconnection, three regimes for the skyrmion dynamical behavior were observed: skyrmion annihilation at the system’s borders, skyrmion motion along non-circular trajectories moving from one disk to the other alternating its position between the two disks, and skyrmion rotation inside only one disk. On the other hand, the variety of dynamical regimes of skyrmions displacing in asymmetric systems such as interconnected nanodisks is enriched if several skyrmions are nucleated in a more complex STNO device. Indeed, due to the skyrmion–skyrmion interaction [39], in addition to the effective forces created by the system’s borders and spin-transfer torque (STT), there is an effective repulsive force that the skyrmions exert on each other [11,39,40,41]. Therefore, one can expect that the dynamical properties of this double skyrmion lying in an interconnected nanodisk system present differences concerning the motion of the single skyrmion case [38]. In this work, we study the magnetization dynamics in a double-disk geometry with one skyrmion at each disk axis as an initial condition. Our results evidence the appearance of four main regimes depending on the geometry and current: stagnation points, oscillatory motion, and total and partial skyrmion annihilation. Indeed, due to the complex balance between the forces responsible for the skyrmion motions, we observe that when the disks present a large superposition, even for large values of electric currents, the skyrmions move until they stop in a stable position in the disks, the stagnation point. In contrast, oscillatory states are obtained for the intermediate values of inter-disk connection. Finally, the annihilation states appear when the disks are weakly connected, and the skyrmions are under the action of large electric currents.

This manuscript is organized as follows: the theoretical model is briefly described in Section 2. In Section 3, the numerical results are performed and characterized. In particular, we carefully analyzed these four regimes and provided a schematic representation of the dynamical states as a function of the geometry and current. The conclusions are presented in Section 4.

## 2. Theoretical Model

**Figure 1:**
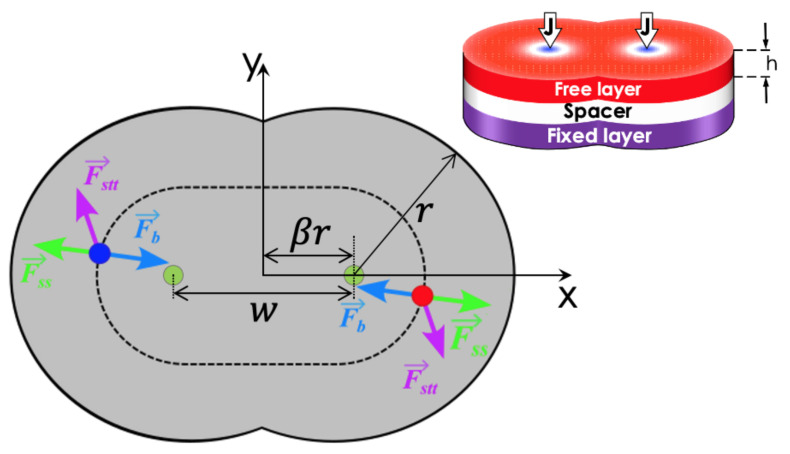
Schematic representation of the double-disk system, with two skyrmions at the positions denoted by the red and blue dots. At the center of each disk, one electrode allows the injection of a polarized current. The forces are also illustrated with lines in green that depict the interactions between the skyrmions, in blue to denote the restoration forces due to the borders, and in burgundy to depict the forces due to the STT.

We consider a typical STNO device consisting of three stacked layers. The top layer is composed of a soft magnetic material free to orient the magnetization according to the system’s condition. The bottom layer is a hard ferromagnetic material whose magnetization is perpendicular to the interfaces. Finally, the intermediate layer separates both ferromagnetic structures. It consists of a thin non-magnetic slab to induce into the soft magnetic layer a high anisotropy and/or DMI strong enough to create two skyrmions. The system under study is a double-interconnected-disk geometry, whose description is given by three main parameters: the radius *r*, the electrode–electrode distance w=2rβ, and the disk thickness h=0.6 nm. To ensure that the skyrmion is a stable configuration in the disks, we consider that the radius of each disk is a little larger than the minimum value that ensures skyrmion stabilization, that is, r=40 nm [25]. In the center of each disk, one electrode allows the injection of a spin-polarized current through the disk axis. The setup of the problem is given in the panel of Figure 1.

To describe the reduced magnetization dynamics of the free layer, we employed the Landau–Lifshitz–Gilbert–Slonczewski (LLGS) equation

(2) $$\frac{\partial \vec{m}}{\partial t}=-\gamma \vec{m}\times {\vec{H}}_{\mathrm{eff}}+\alpha \left(\vec{m}\times \frac{d\vec{m}}{dt}\right)+{\vec{T}}_{\mathrm{STT}},$$

where γ is the gyromagnetic factor, ${\vec{H}}_{\mathrm{eff}}=-\left(1/\mu_{0}M_{s}\right)\delta E/\delta \vec{m}$ is the effective field. Concerning energies, *E* is the total energy density obtained by adding the exchange ($E_{x}$), magnetostatic ($E_{m}$), anisotropy ($E_{a}$), and DMI ($E_{d}$) terms. In addition, α is a dimensionless damping parameter, and ${\vec{T}}_{\mathrm{STT}}$ is the spin-transfer torque term due to the spin-polarized current, and can be cast in the form

(3) $${\vec{T}}_{\mathrm{STT}}=\frac{J}{\xi }\vec{m}\times \left({\vec{m}}_{P}\times \vec{m}\right)-\zeta \frac{J}{\xi }\vec{m}\times {\vec{m}}_{P},$$

where *J* is the current density, $\xi =2\mu_{0}\left|e\right|hM_{s}/\left(\gamma ћP\right)$. In an aforementioned equation, $M_{s}$ is the saturation magnetization, *e* is the electron charge, $\mu_{0}$ is the vacuum permeability, and h represents the dimension of the spacer or free layer. Additionally, *ћ* is the Planck constant, ${\vec{m}}_{P}$ is the polarization vector, *P* is the polarization, while ζ is the magnitude of the out-of-plane torque relative to the in-plane torque.

We remark that Equation (2) is solved through micromagnetic simulations with Mumax3 [42], which is a well-known code that allows good control over the considered nanoparticle. The results on several systems obtained with this micromagnetic code show good agreement with experiments. The magnetic parameters describing the free layer are a stiffness constant A=15 pJ/m, a saturation magnetization $M_{s}=5.8$ MA/m, a uniaxial anisotropy given by K=0.8 MJ/m^3^, and a damping constant α=0.01. This set of parameters allows the nucleated skyrmions to be far from the range for which elliptical instability is generated, as shown in Ref. [43], and additionally allows the nucleation of a skyrmion pair into the system [25]. We also consider that the non-magnetic-metal induces a DMI of magnitude D=3.0 mJ/m^2^ in the free layer. As stated before, the magnetization of the bottom layer is fixed along the direction perpendicular to the nanodisk interface, that is, ${\vec{m}}_{P}=-\hat{z}$. Finally, the non-magnetic material is described by the parameters ζ=0.1 and P=0.3. In our simulations, we consider a current density *J* varying from $J=1\times 10^{10}$ A/m^2^ to $2.5\times 10^{12}$ A/m^2^. The initial conditions for all simulations consist of two skyrmions, each nucleated around the electrodes, as shown in Figure 1.

From the theoretical point of view, the skyrmion dynamics in STNO nano-devices can also be studied by considering that the skyrmion does not deform during its motion. In this case, its trajectory can be determined by following the position of its center over time. This rigid body approximation allows using the Thiele equation [44] $\vec{G}\times \vec{V}+D\cdot \vec{V}+\vec{F}=0,$ for determining the skyrmion position. Here, $\vec{G}=G_{z}\vec{z}$ is the gyrocoupling vector, D is the dissipation dyadic tensor, and $\vec{V}=\dot{\vec{R}}$. The electric current injected into the system is responsible for the STT force, whose magnitude is proportional to the current density *J*. In this way, the effective force acting on the skyrmion is $\vec{F}={\vec{F}}_{b}+{\vec{F}}_{\mathrm{STT}}$, with ${\vec{F}}_{b}$ representing the restoration force that the border of the system exerts on the skyrmion, and ${\vec{F}}_{\mathrm{STT}}$ is the STT force. Both forces, ${\vec{F}}_{b}$ and ${\vec{F}}_{\mathrm{STT}}$, depend on $\vec{R}$.

Although the several interactions acting on the skyrmions can deform their shapes, we can use the Thiele approach to obtain qualitative descriptions of the skyrmion dynamics in the considered structure. Therefore, for such a description of the two-skyrmion propagating under the action of an electric current in the interconnected disk system, we make use of previous results regarding effective forces [31,37,38] acting on the skyrmions. Firstly, the skyrmions are under the action of a force coming from the system’s borders. In this case, due to the asymmetry presented by the considered system, we have that [37] ${\vec{F}}_{b}=-F_{b}^{\rho }\hat{\rho }-F_{b}^{\theta }\hat{\theta }$, where $F_{b}^{\rho }$ and $F_{b}^{\theta }$ are, respectively, the orthogonal and tangent to the borders components of the force. The functional form of the ${\vec{F}}_{b}$ in the considered geometry is more complex as compared to that for a circular STNO device, which have azimuthal symmetry and the tangential component to the force vanishes. In addition to the border effects, the skyrmions are under the action of two STT forces ${\vec{F}}_{\mathrm{STTL}}$ and ${\vec{F}}_{\mathrm{STTR}}$, where ${\vec{F}}_{\mathrm{STTL}}$ and ${\vec{F}}_{\mathrm{STTR}}$ are the STT force due to the electric current in the left and right disk, respectively. Finally, there is a skyrmion–skyrmion interaction with a repulsive force [39] ${\vec{F}}_{R}$, as shown in Figure 1.

The described phenomenology evidences that the two-skyrmion system lying in the interconnected disks presents different static and dynamical behaviors compared to a single or double-disk structure hosting just one skyrmion. In fact, because of the absence of an azimuthal symmetry and the existence of a skyrmion–skyrmion interaction, the equilibrium position of both skyrmions at zero current is not exactly at the electrodes in the center of each disk. Indeed, the equilibrium positions depend on the interconnection between the disks, which affects the skyrmion–skyrmion distances. In the same direction, if the skyrmions are under the action of a STT originated on the spin-polarized current injected through each electrode, their motion should follow trajectories that depend on the STNO geometry, the current density, and the distance between the skyrmions. Thus, we can conclude that the motion of each skyrmion is determined by four contributions, which give place to the emergence of a plethora of dynamical regimes not observed in the one-disk [37] or one-skyrmion counterparts [38].

A complete analysis of the dynamical regimes of two skyrmions in the interconnected two-disk system can be performed through micromagnetic simulations that enable the exploration of the effect of both the disk’s connection characterized by β and the external electric current density denoted by *J*. The following section presents our main results and discussions based on the theoretical model.

## 3. Results

### 3.1. Static Behavior

**Figure 2:**
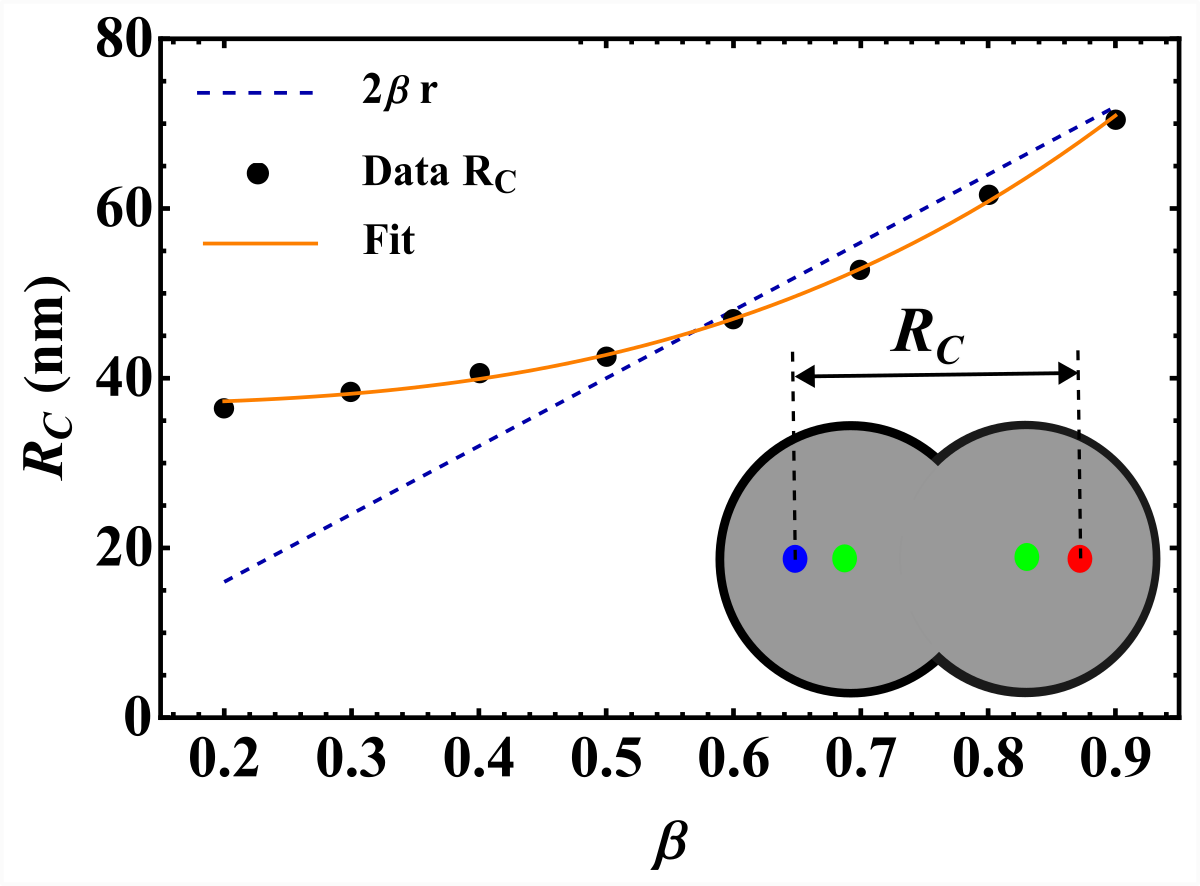
Distance between the skyrmion–skyrmion centers at equilibrium, $R_{c}$, as a function of the nanodisk interconnection β. The dashed line represents the distance between both electrodes, *w*. The inset is a view of the simulated system. Green dots represent the electrodes, while blue and red dots represent the skyrmions nucleated in each disk, separated by the distance $R_{c}$.

As stated before, due to the absence of circular symmetry, the effective force exerted on each skyrmion by the interconnected double-disk borders has a complex structure. In addition, the skyrmion–skyrmion interaction depends on the distance between them [39]. Therefore, the competition between these interactions determines the static behavior of the skyrmions. The restoration force on the skyrmions (shown in blue in Figure 1) has the form ${\vec{F}}_{R}=-\nabla U$, where *U* is the potential due to finite boundaries in the double-disk. Therefore, in the absence of STT forces, the competition between the restoration force and the inter-skyrmion interaction (shown in blue and burgundy in Figure 1) influences the equilibrium position of each skyrmion. In this context, we have analyzed the equilibrium position of the skyrmions as a function of the nanodisk interconnection. The obtained results are described in terms of the distance between the skyrmion centers $R_{c}$, and are presented in Figure 2, where the dashed line represents the disk center-to-center distance, *w*. One may notice a critical value $\beta =\beta^{*}\approx 0.6$ that limits two different behaviors of $R_{c}$ as a function of β. For β>0.6, the distance between the skyrmion’s centers increases monotonically with β, indicating that both skyrmions almost do not interact with each other since their centers are in the vicinity of the electrode positions. Nevertheless, one observes that, for $\beta <\beta^{*}$, the skyrmion–skyrmion interaction dominates, and $R_{c}>w$. It is interesting to mention that $R_{c}$ follows a cubic power law, $R_{c}=a+b\beta^{3}$, with a=36.91 and b=46.69.

### 3.2. Dynamic Regime

A current density J>0 is injected into each electrode to find the different propagation regimes. The spin-transfer torque creates new forces on the skyrmions, hereafter referred to as STT forces, as explained in Ref. [38] and illustrated in green vectors in Figure 1. Several simulations were performed during a time τ=100 ns for $5\times 10^{10}\;A/m^{2}<J<5\times 10^{12}\;A/m^{2}$. The STT forces induce different skyrmion propagation regimes as a function of β and *J*. These regimes are classified as stagnation, oscillatory motion, and annihilation states.

**Figure 3:**
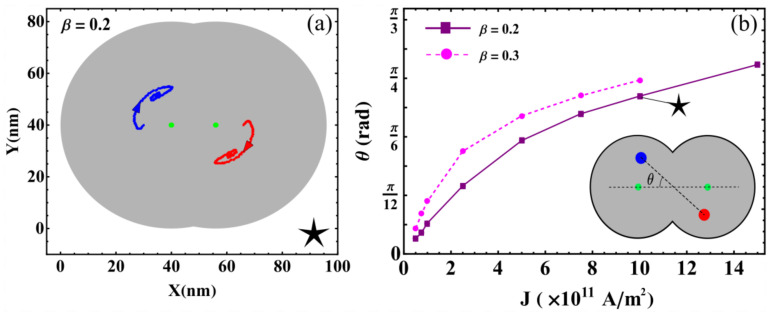
(**a**) Illustration of the skyrmion trajectories until stagnation. Green dots represent the electrodes. (**b**) Angle that characterizes the equilibrium position of both skyrmions as a function of *J* for β=0.2 (purple squares) and β=0.3 (magenta dots). In the inset, the red and blue solid dots represent the skyrmion positions for the last current value for which stagnation points are reached. The star shows the parameters used to obtain the skyrmion trajectory depicted in figure (**a**).

**Figure 4:**
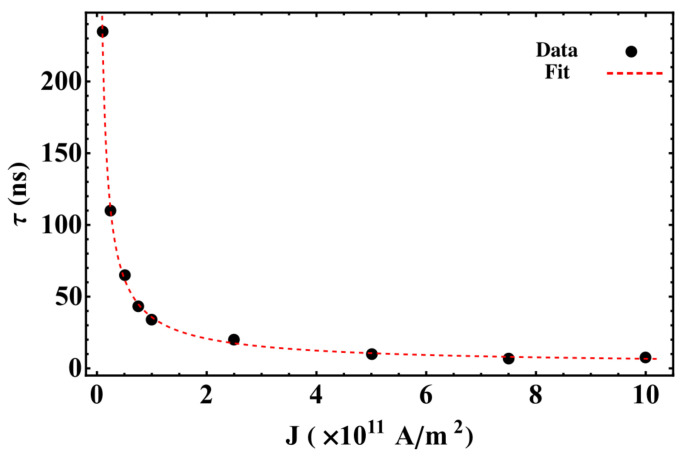
Transient time needed by the skyrmions to reach the stagnation point as a function of the current density for interconnected disks with β=0.2. Black dots present the results obtained by micromagnetic simulations and red-dashed line is a fit given by $a+b/J^{P}$, with a=1.8, b=34, and p=0.8.

**Figure 5:**
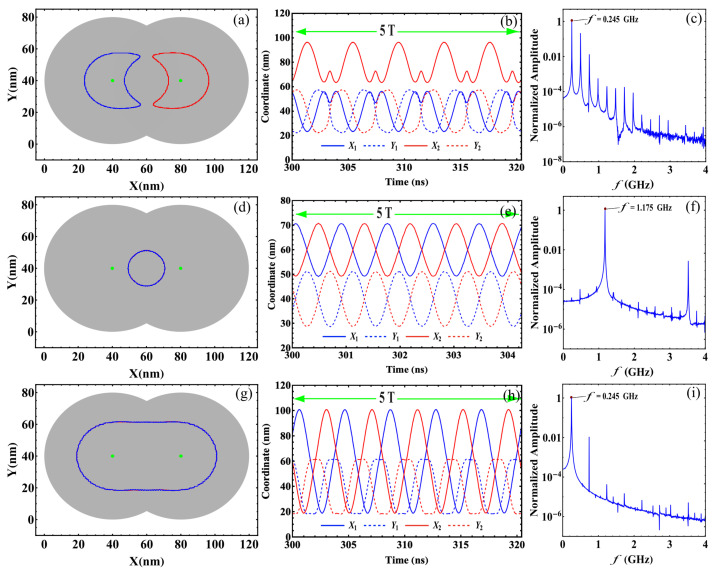
Three examples of oscillatory motions obtained showing the skyrmion trajectories in the phase space, the time series of the four components, and the amplitude as a function of the frequency of the corresponding global fast Fourier transform of one skyrmion. For all cases, we fixed the geometric parameter at β=0.5 and varied the current: (**a**–**c**) $J=2.5\times 10^{10}\;A/m^{2}$, (**d**–**f**) $J=5\times 10^{10}\;A/m^{2}$ and (**g**–**i**) $J=2.5\times 10^{12}\;A/m^{2}$.

**Figure 6:**
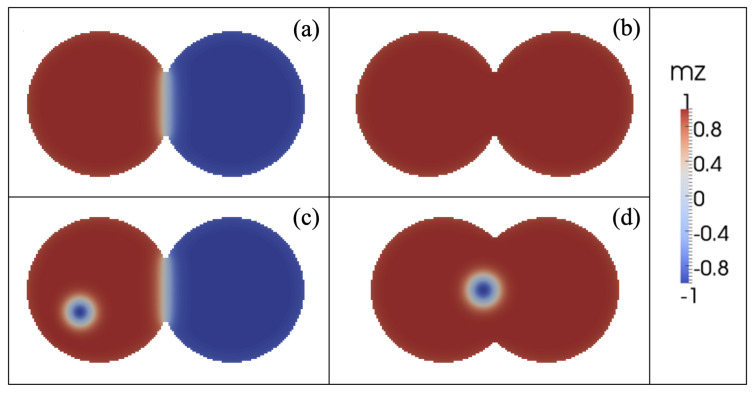
Snapshot of the magnetic configurations after annihilation at t=0.5 μs for β=0.9 and (**a**) $J=2.5\times 10^{11}$ A/m^2^, (**b**) $J=2.5\times 10^{12}$ A/m^2^, (**c**) $J=1.0\times 10^{10}$ A/m^2^, and (**d**) β=0.7 and $J=2.5\times 10^{12}$ A/m^2^. Panels (**a**,**b**) represent total annihilation processes, while panels (**c**,**d**) illustrated partial annihilation behaviors.

1. *Convergence to stagnation*: In this regime, the balance between the restoration, STT, and inter-skyrmions forces allows each skyrmion to reach stagnation points after a brief transient. This behavior was previously reported in asymmetric circular-STNO devices [38] and is illustrated in Figure 3a. This regime appears for small *J* and β, and is characterized by the stagnation angle shown in Figure 3b, which illustrates this angle that characterizes the equilibrium position of both skyrmions as a function of *J* for β=0.2 and 0.3. From this figure, we observe that θ increases with *J*, reaching a critical value, $\theta_{c}$, that depends on β. For $\theta >\theta_{c}$, no more stagnation points were obtained for each β. We can notice that θ obeys different power laws, as a function of *J* depending on β. For instance, at β=0.2, we find that $\theta ∼\sqrt[{3}]{J}$. The stagnation points can be also analyzed from the transient time, τ, necessary so that the skyrmions may reach their equilibrium positions. In this case, we studied the behavior of τ as a function of the current density for an interconnected disk with β=0.2. The obtained results are depicted in Figure 4, where we notice a decrease in the transient time as the electric current increases. This behavior can be explained by the low skyrmion precession velocity due to the unbalanced forces for small current densities. That is, the increase in the skyrmion velocity as a function of the current density yields a smaller time by which the skyrmions reach their equilibrium positions.
2. *Oscillatory motion*: In this regime, both skyrmions keep precessing following different types of trajectories. Three examples of these trajectories are shown in Figure 5. This behavior occurs for small β and $J>1\times 10^{12}$ A/m^2^, resulting from the strength of the STT force, which creates a restoration force that prevents the annihilation of skyrmions at the borders of the double-disk structure.
  The specific trajectory followed by the skyrmions depends on *J* and β and results from the balance between the restoration, STT, and skyrmion–skyrmion interaction forces. Examples of the dynamical behaviors for $J=2.5\times 10^{10}\;A/m^{2}$, $J=5\times 10^{10}\;A/m^{2}$, and $J=2.5\times 10^{12}\;A/m^{2}$ (from top to bottom) at β=0.5 are depicted in Figure 5. For each value of the electric current, we show the trajectory in the phase space, the time series of the component, and the corresponding global fast Fourier transform (FFT) for one skyrmion calculated as follows $\left|F\right|=\sqrt{F_{x}^{2}+F_{y}^{2}}$, where $F_{\zeta }$ is the FFT of the ζ-component. Note that it is only needed to present the FFT of one skyrmion, since the other is identical. In the upper panels, at $J=2.5\times 10^{10}$ A/m^2^, we can observe that each skyrmion precesses around its electrode. Moreover, the time series show that the trajectories are anti-synchronized because when the *x* component of one of the skyrmions is at its maximum value, the other is at its minimum value. The same behavior is observed for the *y* components. From the FFT, it is clear that states are multi-periodic, with the main peak at f=0.245 GHz. The intermediate panels show the trajectories for $J=5\times 10^{10}\;A/m^{2}$. Here, we can observe that the skyrmion motions are periodic with the principal frequency at f=1.175 GHz, such that they are confined to the region between the electrodes. Again, the trajectories are anti-synchronized in the *x* and *y* components. Finally, the bottom panels of Figure 5 show periodic motions for $J=2.5\times 10^{12}$ A/m^2^, with the principal frequency at f=0.245 GHz. In contrast to the previous cases, the phase space plot shows that both skyrmions rotate around the region outside the electrodes, with a phase shift between them, as shown in the time series.
3. *Annihilation*: Annihilation regimes are characterized by at least one skyrmion annihilation after a transient time. We highlight here that two different final states are related to this regime. In the first one, called total annihilation, both skyrmions annihilate, and the disks exhibit anti-parallel (Figure 6a) or parallel (Figure 6b) saturated magnetization, and if the current is high enough, once the borders of the skyrmion touch the edges of the disk, the skyrmion is quickly annihilated. A different behavior corresponds to what we call partial annihilation, which consists of the annihilation of just one skyrmion, and the other keeps oscillating around the electrode (Figure 6c), or close to the center of the double-disk system (Figure 6d). It is important to notice that if a low current is injected during the annihilation processes (global or partial), it is possible to create a domain wall at the center of the system that creates two domains with anti-parallel magnetization (Figure 6a,c). However, as in figures, for higher currents, such as $J=2.5\times 10^{12}$ A/m^2^. Figure 6b,d, creating a domain wall is no longer possible, and the magnetization in both disks after the annihilation process is parallel. Videos of the different annihilation processes are included as Supplementary Materials.

**Figure 7:**
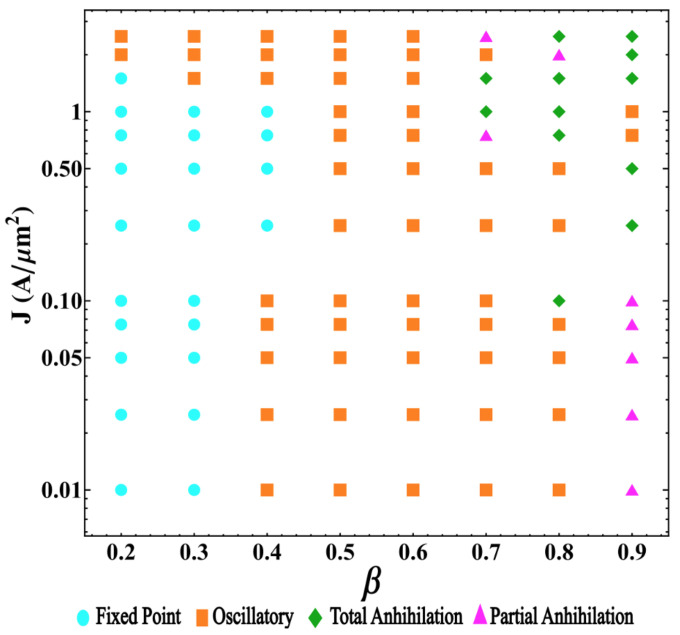
Two-dimensional state diagram of the skyrmion dynamics as a function of β and *J*.

All the numerical obtained results are included in the two-dimensional diagram of the skyrmion dynamical states as a function of β and *J*. Figure 7 illustrates the obtained results, where the cyan dots, orange squares, pink triangles, and green diamonds represent, respectively, the stagnation, oscillatory, and partial and total annihilation states. This state diagram (SD) results from the complex balance between the forces responsible for these four regimes. We can observe that small values of β and large values of *J* yield stagnation point solutions for the considered range of parameters. In contrast, the oscillatory states are predominant for intermediate values of β. Finally, the annihilation states appear for large values of both β and *J*. The obtained results for β≳0.65 and $J≳0.2\times 10^{12}$ A/m^2^ reveal some uncertainty in the final state, originating from the complex behavior of the interactions that both skyrmions are subject to. Therefore, in this range of parameters, results point to a possible chaotic behavior of the skyrmion dynamics. Nevertheless, because chaotic regimes demand the analysis of excessively long time series for each set of parameters, the study of the chaotic behavior of the dynamical regimes of skyrmion pair in double-disks systems demands further analysis that will be part of future projects.

## 4. Conclusions

In this work, we study the behavior of two skyrmions propagating in an STNO device with an interconnected disk geometry under the effect of an electric current density. Concerning the dynamics, we obtained four main regimes: stagnation points, periodic motion, and two types of skyrmion annihilation: partial and total. We observe that the angle between the skyrmions for stagnation point solutions follows power laws as a function of the electric current and the geometric parameters. The oscillatory regime involves several dynamical behaviors, some of which were illustrated in this work. In particular, we found periodic and multi-periodic states. In this case, the skyrmions can oscillate inside one disk subsystem or around the two interconnected disks depending on the electric current and geometrical parameters. In addition, some of the skyrmions motions are anti-synchronized. Furthermore, for large distances between the electrodes and high currents, we observe the existence of different annihilation processes that involve the annihilation of a unique or both skyrmions. Videos illustrating these states are included as Supplementary Materials. Our results allow us to produce a two-dimensional SD of the skyrmion’s dynamics as a function of the disk’s connection, β, and the electric current density, *J*. We can observe that for β<0.3 and $J<10^{12}$ A/m^2^, we obtain skyrmions that stop at some position inside the disk, reaching stagnation points. For β in the range between 0.4 and 0.7, the oscillatory regime is obtained. Furthermore, finally, for β<0.8 and $J>0.5\times 10^{12}$ A/m^2^, a partial annihilation of skyrmions is obtained. We expect that our work can motivate and guide new experiments that can confirm these results and contribute to the development of new applications based on skyrmion dynamics.

## Data Availability

All data that support this study are included within the article and in the supplementary file.

## Supplementary Materials

The following supporting information can be downloaded at: https://www.mdpi.com/article/10.3390/nano12213793/s1, Video S1: Stagnation point at β=0.2 and $J=1\times 10^{12}$ A/m^2^. Video S2: Oscillatory state at β=0.5 and $J=5\times 10^{10}$ A/m^2^. Video S3: Oscillatory state at β=0.5 and $J=2.5\times 10^{12}$ A/m^2^. Video S4: Annihilation state at β=0.9 and $J=2.5\times 10^{12}$ A/m^2^. Video S5: Annihilation state at β=0.9 and $J=2.5\times 10^{11}$ A/m^2^. Video S6: Annihilation state at β=0.9 and $J=2.5\times 10^{10}$ A/m^2^. Video S7: Annihilation state at β=0.7 and $J=2.5\times 10^{12}$ A/m^2^.

## Abbreviations

The following abbreviations are used in this manuscript:

DMI	Dzyaloshinskii–Moriya Interaction
STNO	Spin Torque Nano-Oscillator
STT	Spin-Transfer Torque
LLGS	Landau–Lifshitz–Gilbert–Slonczewski
J	Electric Current Density
FFT	Fast Fourier Transformation
SD	State Diagram
